# Nonpharmacological pain management approaches among U.S. construction workers: A cross‐sectional pilot study

**DOI:** 10.1002/ajim.23630

**Published:** 2024-06-20

**Authors:** Aurora B. Le, Abas Shkembi, G. Scott Earnest, Elizabeth Garza, Douglas Trout, Sang D. Choi

**Affiliations:** ^1^ Department of Health Behavior, School of Public Health Texas A&M University College Station Texas USA; ^2^ Department of Environmental Health Sciences, School of Public Heath University of Michigan Ann Arbor Michigan USA; ^3^ Office of the Director National Institute for Occupational Safety and Health Washington District of Columbia USA; ^4^ Department of Global and Community Health, College of Public Health George Mason University Fairfax Virginia USA; ^5^ Department of Occupational & Environmental Safety & Health University of Wisconsin – Whitewater Whitewater Wisconsin USA

**Keywords:** boosted regression tree, construction, nonpharmacological pain management, opioids, workers

## Abstract

**Background:**

U.S. construction workers experience high rates of injury that can lead to chronic pain. This pilot study examined nonpharmacological (without medication prescribed by healthcare provider) and pharmacological (e.g., prescription opioids) pain management approaches used by construction workers.

**Methods:**

A convenience sample of U.S. construction workers was surveyed, in partnership with the U.S. National Institute for Occupational Safety and Health (NIOSH) Construction Sector Program. Differences in familiarity and use of nonpharmacological and pharmacological pain management approaches, by demographics, were assessed using logistic regression models. A boosted regression tree model examined the most influential factors related to pharmacological pain management use, and potential reductions in use were counterfactually modeled.

**Results:**

Of 166 (85%) of 195 participants reporting pain/discomfort in the last year, 72% reported using pharmacological pain management approaches, including 19% using opioids. There were significant differences in familiarity with nonpharmacological approaches by gender, education, work experience, and job title. Among 37 factors that predicted using pharmacological and non‐pharmacological pain management approaches, training on the risks of opioids, job benefits for unpaid leave and paid disability, and familiarity with music therapy, meditation or mindful breathing, and body scans were among the most important predictors of potentially reducing use of pharmacological approaches. Providing these nonpharmacological approaches to workers could result in an estimated 23% (95% CI: 16%–30%) reduction in pharmacological pain management approaches.

**Conclusion:**

This pilot study suggests specific factors related to training, job benefits, and worker familiarity with nonpharmacological pain management approaches influence use of these approaches.

## 1 INTRODUCTION

In the United States (U.S.) the construction industry, both historically and in the present day, has some of the highest rates of injury, illness, and fatality rates across all occupations. In 2020, workers in construction had 41,400 nonfatal workplace injuries due to falls, slips, and trips—an increase from 94.8 cases to 127.2 cases per 10,000 full‐time workers compared to the prior year.^1^ From the same year, the U.S. Bureau of Labor Statistics (BLS) also measured incidence rates of nonfatal injuries or illnesses resulting in days away from work due to overexertion and found that 93% of construction laborers routinely engage in a medium (54.0% construction vs. 28.2% all civilian workers) or heavy strength of exertion (38.8% construction vs. 8.9% all civilian workers) daily.^1^ This resulted in a 2020 incidence rate of overexertion and bodily reaction of 48.3 cases per 10,000 full‐time workers, making them highly susceptible to musculoskeletal disorders (MSDs).^2^ The physically demanding nature of construction work may not only yield short‐term consequences, such as missed work days, but also can result in the sequelae of acute pain and MSDs, leading to subsequent long‐term chronic pain.^3^

With the extent of chronic pain experienced by construction workers, the opioid epidemic has disproportionately affected the industry. As of 2021, there have been 645,000 deaths nationwide from an overdose involving any opioid, prescription (licit) and illicit, in two decades; in 2020 over 250 lives were lost from overdoses every day.^4^ Construction workers have been regularly prescribed opioids—even for minor injuries—increasing their susceptibility to misuse and overdose.^5^ State‐level studies have found that construction workers are six to seven times more likely to die of an opioid overdose than workers in other professions, from licit (e.g., oxycodone and hydrocodone preparations) and illicit sources (such as heroin, fentanyl).^6^ From 2011 to 2016, construction workers experienced 15% of all worker overdose deaths despite representing slightly greater than 5% of the workforce. Researchers have identified high rates of workplace injuries as a potential leading cause of the high prevalence of opioid use among construction workers.^7^, ^8^, ^9^, ^10^

Considering the ongoing opioid epidemic, there is little information on nonpharmacological pain management approaches used by construction workers and their efficacy. Nonpharmacological pain management are pain therapies, interventions, and approaches that do not involve prescription medication. These include physical therapy, occupational therapy, over‐the‐counter (OTC) medications, chiropractic care, acupuncture/acupressure, meditation, topical ointments, music therapy, cognitive behavioral therapy (CBT), and spiritual healing, among many.^11^, ^12^, ^13^ Healthcare providers have been exploring alternatives to pain management due to the overprescription of opioids.^4^ In a randomized clinical trial in an emergency department, the analgesic effects of a non‐opioid combination (ibuprofen and acetaminophen) were as effective as a single dose opioid in treating acute extremity pain.^14^, ^15^ Additionally, literature demonstrates that non‐Western cultures are more likely than Western ones to use nonpharmacological pain management approaches, due to differing beliefs about Western medicine, individualism versus communalism, and spiritual and cultural approaches to pain management.^16^, ^17^, ^18^ With 37% of the construction industry composed of racial minorities (including Hispanic/Latino, non‐Hispanic (NH) Black, NH Asian)—compared to 23% of the U.S. general workforce—nonpharmacological pain management approaches are may potentially be culturally relevant to this sector.^1^

To our knowledge, no study has been conducted in the U.S. to determine the nonpharmacological pain management approaches being used by construction workers. We conducted a cross‐sectional pilot study among a sample of construction workers with the goal of answering the following exploratory research questions: (1) with what nonpharmacological pain management approaches are construction workers familiar? and (2) what nonpharmacological pain management approaches do construction workers use? This study provides self‐reported data on what construction workers use to manage pain. This will help to better inform workplace education and training around pain management, and serve as an evidence base to develop interventions to reduce pain among construction workers.

## 2 MATERIALS AND METHODS

### 2.1 Participants

This cross‐sectional pilot study characterized pain management approaches using self‐reported information from a survey administered by researchers. Pilot study participants were those aged 18 and older, who worked in the U.S. construction industry at the time of survey administration, and were able to provide electronic informed consent in English. Participants were recruited nationally via social media campaigns (such as Twitter/X and LinkedIn), construction industry stakeholders (trade and labor unions, business owners, trade organizations, and the Center for Construction Research and Training [CPWR]), and chain referral sampling (wherein current participants help recruit prospective participants). This study was approved by the University of Michigan Institutional Review Board (Protocol # HUM00222058).

### 2.2 Questionnaire

The survey instrument was adapted from multiple sources, including a study on nonpharmacological pain management approaches used by healthcare workers in Nigeria^19^ and the NIOSH Quality of Worklife (QWL) questionnaire.^20^ The survey did not ask detailed questions about the worker's pain because the focus of the study was not to conduct an intervention; rather the survey explored the participants' familiarity and use of nonpharmacological pain management approaches. Other literature on nonpharmacological pain management has been patient‐focused (e.g., reducing pain during childbirth) rather than worker‐focused. The original draft survey had 165 questions and underwent extensive revisions following subject matter expert reviews to reduce respondent burden, and ensure the final questionnaire was salient in answering the proposed research questions.

Electronic survey administration via Qualtrics (©2022, Provo, UT, USA) occurred from August to November 2022. There were 15 primary questions concerning work‐related pain (with skip‐logic questions and responses that appeared when applicable), location (arms, legs or back within the last 12 months), whether they sought medical care for the pain and if they were prescribed an opioid, participants' familiarity with, and use of, both pharmacological and nonpharmacological pain management approaches, job benefits, and union status.

Participants identified their familiarity with various pharmacological (i.e., any prescription drug prescribed by a healthcare provider) and nonpharmacological (i.e., over‐the‐counter medication, techniques to alleviate pain) pain management approaches by being asked “Which pain management approaches are you aware/familiar with?” or “Which of the following medications (opioids and non‐opioids) are you aware/familiar with?”, selecting from a list of 19 approaches and responding with “Not familiar at all,” “Slight familiar,” “Moderately familiar,” “Very familiar,” or “Extremely familiar.” Participants were considered to be “familiar” with a given approach if they responded with at least “Slightly familiar.” Participants further chose from the same list of 19 approaches when asked their use of various pharmacological and nonpharmacological pain management approaches (“Have you USED any of these treatments to manage the pain you experienced in the last 12 months?”), responding with “Not at all,” “Rarely,” “Occasionally,” “Sometimes,” “Very often.” Participants were considered to have used a given approach if they responded with “Occasionally,” “Sometimes,” or “Very often.”

There were also 15 questions on participant characteristics and demographics including age, weight and height, gender, marital status, education level, employment status, job title/role, years in the construction industry, race and ethnicity, and country of birth.

### 2.3 Statistical analyses

All data cleaning and analyses were done in R v4.0.2 (RStudio, Boston, MA, USA). Descriptive statistics (n, %) were run on all variables. Differences in treatments used for pain management by gender, work experience, education level, union status, race/ethnicity, job title, and job type were examined using simple linear regression and chi‐squared ANOVAs associated with the regression; a two‐tailed *p* < 0.05 was considered statistically significant. A machine learning approach, boosted regression trees (BRT),^21^, ^22^ was further employed to examine the most important predictors of workers using pharmacological pain management approaches. A total of 37 predictors were included in the model, four relating to demographics, ten relating to job benefits, four relating to MSD pain, pain management, and training, and nineteen relating to familiarity with pharmacological and nonpharmacological pain management, among others. The entire set of predictors included in the model are shown in APPENDIX Table A1. The BRT model allows flexibility to observe non‐additive relationships, which mirrors how multiple factors may interact to influence whether a worker used pharmacological approaches for pain management. The BRT model also identifies which predictors are most influential to the outcome. Lastly, it can better handle analyses with smaller sample sizes and a larger set of predictors than traditional regression techniques, making it suitable to handle this study's analyses.

The BRT model grew 1000 trees on a Bernoulli distribution with a learning rate of 0.01 and a tree complexity of 10 (i.e., up to 10‐way interactions of predictors). For any one of the 1000 trees, only a random 50% subset of the predictors (without replacement) were available for selection to introduce randomness into the model. This allows each predictor a chance to be included in the model, preventing a handful of predictors from dominating the model building. The BRT was also trained on a random sample of 75% of participants (without replacement) to minimize overfitting. To determine the most important predictors of the model, the relative influence (RI) of each predictor was calculated by weighting the number of times a predictor was selected in a tree's splitting step and the squared improvement (reduction in error) that the predictor provided to the model, averaged over all trees, and scaled to 100%. A higher RI demonstrates a stronger relationship (positively or negatively) with pharmacological pain management approaches.

To quantify the possible causal effect of important predictors on workers using pharmacological approaches, we utilized a counterfactual method that changes “exposure” status within the model to predict reductions of an outcome due to the exposure change.^23^, ^24^ Traditionally, this counterfactual method related to changing the status of an individual from “exposed” to “unexposed;” in this study, this method relates to changing the status of whether workers are aware of certain nonpharmacological pain management approaches, have certain job benefits, or have received certain trainings.

For example, consider the counterfactual scenario wherein all workers received training on the risks of opioids. The BRT model can then be rerun to predict the number of workers hypothetically using pharmacological approaches under this counterfactual scenario (*N*_*S*_), compared to the observed number of workers using these approaches (*N*_*O*_). We can then estimate the predicted percentage reduction (*R*_*S*_) in workers using pharmacological approaches associated with training on the risks of opioids:

$$R_{S}=\frac{N_{O}-N_{S}}{N_{O}}\times 100\%$$

This estimation can then be replicated for every other relevant predictor to estimate the single effect of each predictor on pharmacological approaches. Combined counterfactual scenarios (e.g., all workers are familiar with meditation and all workers received training on the risks of opioids) can be utilized to estimate the effect of multiple predictors. For these simulations, 1000 bootstrap samples are generated to introduce randomness and variability into the calculation of *R*_*S*_, and the process outlined above is replicated 1000 times. The mean and 95% confidence interval (CI) of *R*_*S*_ from these 1000 bootstrap samples is used as the final estimate of the prediction reduction in workers using pharmacological pain management approaches. Lastly, we considered *i* iterative, counterfactual scenarios that would represent the estimated percent reduction of workers using pharmacological pain management approaches for the best set of *i* variables.

## 3 RESULTS

The study participants (*N* = 195) were primarily male (83%), NH white (80%), and born in the U.S. (96%). Most participants were in a union (83%), had >20 years of work experience (55%), and a high school diploma (or equivalent) (54%). The majority of study participants were hourly workers or in a non‐supervisory role (55%) or laborers/helpers (36%). Of 195 construction workers, 166 (85%) reported experiencing any musculoskeletal pain/discomfort in their arms, legs, or back in the last 12 months (Table 1). Pharmacological pain management approaches were slightly more common (65%) than nonpharmacological approaches (59%). Together, 50% of participants (97) reported using both pharmacological and nonpharmacological pain management approaches in the 12 months. Around 1 in 8 participants (13%) reported using either prescribed or illicit opioids to manage their pain. Of the 85% of participants reporting musculoskeletal pain/discomfort in the last year, 72% reported using pharmacological pain management approaches, including 19% using opioids. The percentage of participants who reported pain in the last year and used nonpharmacological pain management, pharmacological pain management, or opioids were similar across each category (Table 1).

**Table 1:** Participant characteristics and self‐reported pharmacological pain management approaches, overall (*n* = 195) and among those who experienced pain^1^ (*n* = 166), used any nonpharmacological pain management approaches^2^ (*n* = 115), used any pharmacological approaches in the last year^3^ (*n* = 127), or prescribed/illicit opioids^4^ (*n* = 25) in the last year.

Characteristic	N	n (%)	Pain^1^ (%)	Non‐pharm.^2^ (%)	Pharm.^3^ (%)	Opioids^4^ (%)
Overall	195	195 (100%)	166 (85%)	115 (59%)	127 (65%)	25 (13%)
Gender	143					
*Male/man*		119 (83%)	104 (87%)	87 (73%)	100 (89%)	17 (14%)
*Female/woman*		18 (13%)	18 (100%)	18 (100%)	16 (84%)	4 (22%)
*Other gender* a		6 (4%)	6 (100%)	4 (67%)	6 (100%)	1 (17%)
Race/ethnicity	138					
*NH White*		110 (80%)	99 (90%)	84 (76%)	96 (87%)	18 (16%)
*Hispanic*		15 (11%)	13 (87%)	10 (67%)	11 (73%)	0 (0%)
*NH Black or African American*		5 (4%)	4 (80%)	5 (100%)	3 (60%)	1 (20%)
*Other race/ethnicity* b		8 (5%)	7 (88%)	5 (62%)	7 (88%)	1 (12%)
Education status	145					
*High school grad/GED/some college*		78 (54%)	68 (87%)	56 (72%)	67 (86%)	13 (17%)
*Associate's degree or higher*		37 (26%)	33 (89%)	31 (84%)	31 (84%)	5 (14%)
*Trade/vocational training*		26 (18%)	25 (96%)	20 (77%)	21 (81%)	4 (25%)
*Did not complete high school*		4 (2%)	4 (100%)	3 (75%)	4 (100%)	1 (25%)
Job type	141					
*Hourly worker/non‐supervisory role*		77 (55%)	71 (92%)	61 (79%)	67 (87%)	13 (17%)
*Supervisor*		43 (30%)	38 (88%)	31 (72%)	36 (84%)	7 (16%)
*Front‐line supervisor*		21 (15%)	18 (86%)	15 (71%)	16 (76%)	2 (10%)
Job title	139					
*Laborers and Helpers*		50 (36%)	42 (84%)	39 (78%)	42 (84%)	7 (14%)
*Masonry Workers*		25 (18%)	23 (92%)	19 (76%)	20 (80%)	6 (24%)
*Managers*		19 (14%)	16 (84%)	15 (79%)	15 (79%)	4 (21%)
*Equipment Operators*		12 (9%)	11 (92%)	10 (83%)	10 (83%)	2 (17%)
*Carpenters*		11 (8%)	11 (100%)	6 (55%)	11 (100%)	1 (9%)
*Electricians*		7 (5%)	7 (100%)	6 (86%)	7 (100%)	1 (14%)
*Other workers*		15 (10%)	14 (93%)	11 (73%)	12 (80%)	1 (7%)
Work experience	145					
*>20 years*		80 (55%)	73 (91%)	61 (76%)	69 (86%)	13 (16%)
*11‐20 years*		27 (19%)	23 (85%)	18 (67%)	22 (81%)	6 (22%)
*2‐10 years*		31 (21%)	27 (87%)	25 (81%)	25 (81%)	4 (13%)
*1 year or less*		7 (5%)	7 (100%)	6 (86%)	7 (100%)	0 (0%)
In a union	167					
*Yes*		139 (83%)	121 (87%)	96 (69%)	106 (76%)	21 (15%)
*No*		28 (17%)	23 (82%)	14 (50%)	17 (61%)	3 (11%)
Born in the US	145					
*Yes*		139 (96%)	124 (89%)	107 (77%)	117 (84%)	23 (17%)
*No*		6 (4%)	6 (100%)	3 (50%)	6 (100%)	0 (0%)

^a^ Other genders include trans male/men and gender non‐conforming individuals.

^b^ Other race/ethnicities include Native Hawaiians or Pacific Islanders, American Indians or Alaska Natives, Asians, and Multiracial individuals.

Table 2 shows reported musculoskeletal pain and pain management strategies. 84% (136 of 163) of respondents said that pain and discomfort made it difficult to do either work/home activities and 26% (41 of 159) missed work days because of pain. Most of this pain and discomfort was reported as caused by work‐related activities (69%). Almost half of respondents with pain and discomfort saw a physician because of the pain (43%), and around 1 in 3 (31%) of those respondents were subsequently prescribed an opioid by the physician. The most common pain management approach participants reported using was OTCs (81%). Over 1 in 10 workers reported taking pain medications prescribed for someone else (12%), and only 9% of workers told their supervisor about the pain or filed workers' compensation claims. Nearly a third of workers (30%) reported using nonpharmacological approaches. The most common strategies were traditional (cold and heat, stretching, physical activity) and nontraditional (alcohol consumption, marijuana use, kratom). While most workers were in a union (83%), nearly three in four workers reported having no paid sick leave (74%), and more than half reported no unpaid leave (57%) (Table 2). Not having paid disability leave was common (55%), and most workers (88%) reported having no on‐site medical care (nursing visits, triage treatments).

**Table 2:** Reported musculoskeletal pain, pain management strategies and job benefits

Characteristic	*N*	*n* (%)
Pain discomfort in last year	195	166 (85%)
*Made difficult to do activities?*	163	136 (84%)
*Caused by work activities?*	165	114 (69%)
*Missed workdays because of pain?*	159	41 (26%)
*Seen physician for pain?*	159	68 (43%)
*Prescribed an opioid by physician?*	68	21 (31%)
Training on risks of opioids	179	99 (53%)
Pain management strategies	166	
*Take over‐the‐counter medications*		134 (81%)
*Go to health practitioner*		64 (39%)
*Other strategies* a		49 (30%)
*Stop doing task that caused pain*		35 (21%)
*Take time off work*		27 (16%)
*Take pain medications prescribed to someone else*		20 (12%)
*Tell supervisor about pain/file worker's compensation*		15 (9%)
Job benefits		
*No onsite nursing visits/treatments*	163	144 (88%)
*No paid sick leave*	165	119 (74%)
*No EAP* b (*mandatory referral only)*	157	115 (73%)
*No paid vacation*	164	96 (58%)
*No unpaid leave*	153	88 (57%)
*No paid disability leave*	166	98 (55%)
*No EAP* (*personal and work‐related issues)*	164	76 (46%)
*No onsite first aid*	159	51 (32%)
*No health insurance*	168	7 (4%)

^a^ Other pain management strategies included alcohol consumption, marijuana use, ice and heat therapy, stretching, or physical activity, among others.

^b^ EAP, employee assistance program. No EAP means this workplace benefit/resource is not available to the workers as an option. Issues that EAP would typically address (e.g., mental health, substance use disorder treatment) are by mandatory referral only.

**Figure 1:**
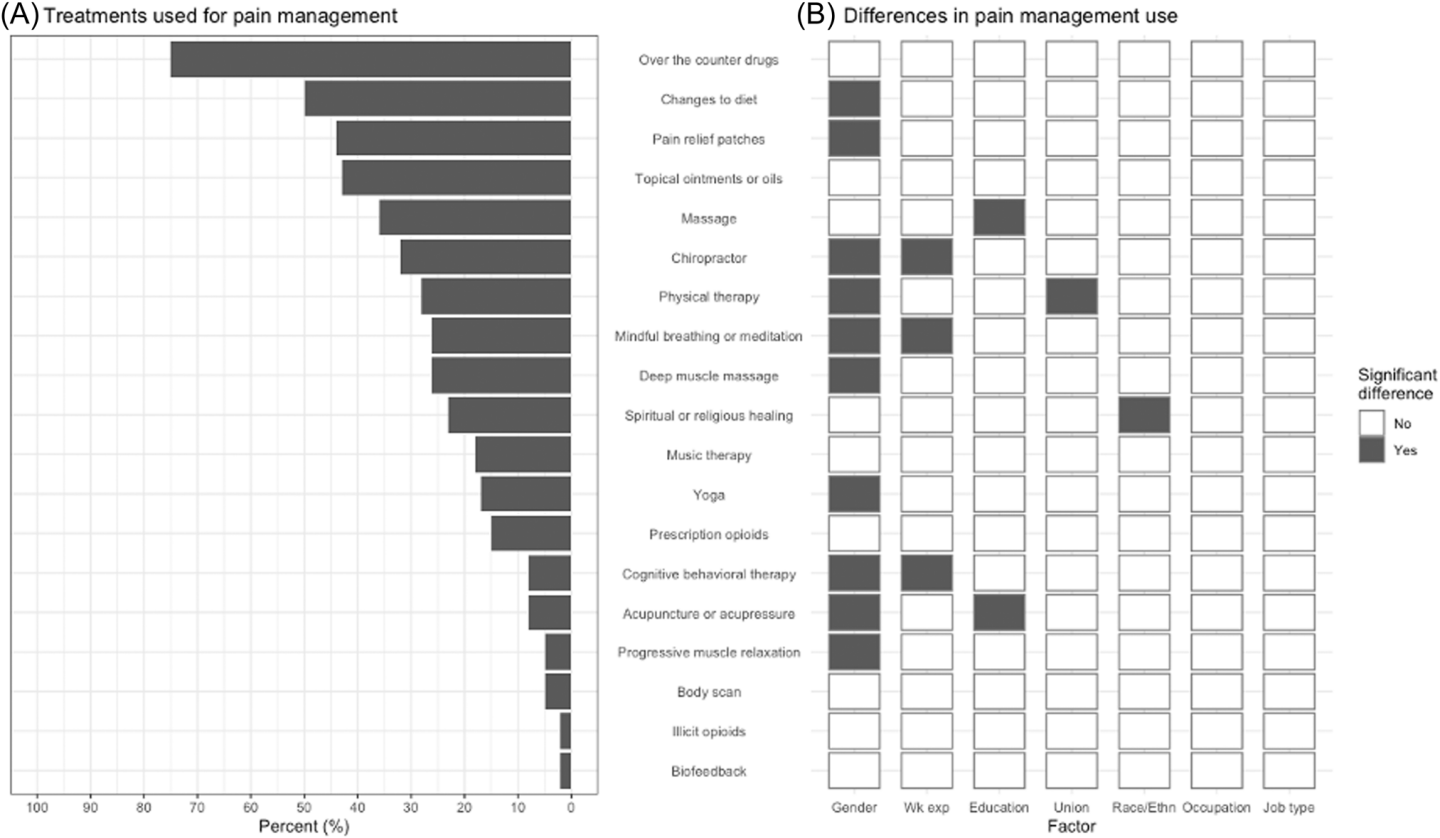
(A) Percentage of respondents who self‐reported treatments used for pain management (*N* = 148‐153). Note that “use” is considered for responses “Occasionally,” “Sometimes,” and “Very Often.” Note: alcohol consumption, marijuana use, Kratom, and stretching/exercising were common other treatments used for pain management. (B) Significant differences (gray box) observed from a simple linear regression model in treatments used by various participant characteristics (from left to right: gender, work experience, education level, union status, race/ethnicity, occupation/job title, and job type [e.g., hourly employee]).

The most commonly used nonpharmacological pain treatments (those used “Occasionally,” “Sometimes” or “Very often”) for pain management were OTC medications (75%), changes to diet (50%), and pain relief patches (44%). The least commonly used nonpharmacological treatments were progressive muscle relaxation (5%), biofeedback (2%), and illicit opioids (2%) (Figure 1). Participants were the least familiar with biofeedback (84%), progressive muscle relaxation (80%), illicit opioids (76%), CBT (71%), and music therapy (66%) (APPENDIX FIGURE A1). There were significant differences in treatments used for pain management by gender, work experience, education, union status, and race/ethnicity. Compared to males, females had higher odds of using progressive muscle relaxation (odds ratio [OR] 11.7, 95% CI: 1.8–75.8), using CBT (OR 9.4, 95% CI: 2.6–33.8), and using acupuncture or acupressure (OR 7.2, 95% CI: 1.9–27.1). Doing yoga, changing diet, using mindful breathing or meditation, deep muscle massages, chiropractic, and physical therapy were also significantly more frequent in females than males Compared to workers with >20 years of work experience, workers with 2–10 years of experience had higher odds of using CBT (OR 7.5, 95% CI 1.8–31.2) and of using mindful breathing or meditation (OR 5.3, 2.1–13.0) to manage their pain. Compared to workers with an associate degree or higher, workers with a high school diploma/GED/some college used massages, acupuncture or acupressure less frequently to manage their pain. Non‐un‐ionized workers used physical therapy less frequently than those in a union. NH Black workers and workers of other races/ethnicities reported using spiritual/religious healing to manage their pain considerably more than did NH White workers (OR 6.8 and 4.5 respectively).

Of the 37 predictors in the BRT model, 12 demonstrated a relative influence (RI) above the 2.7% randomness threshold (APPENDIX TABLE A1). The three most influential predictors of ever using pharmacological pain management approaches were whether pain made work/home activities difficult to complete (RI = 19.6%), education level (RI = 9.1%) and familiarity with prescription opioids (RI = 6.8%). Other notable predictors of ever using pharmacological pain management approaches were training on risks of opioids (RI = 5.8%), job benefits that included unpaid leave (RI = 3.9%), and paid disability (RI = 3.2%), When considering the most important predictors of ever using nonpharmacological pain management approaches, familiarity with mindful breathing/meditation (RI = 4.3%), familiarity with music therapy (RI = 3.9%) and familiarity with body scans (RI = 3.5%) demonstrated the highest relative influence.

**Figure 2:**
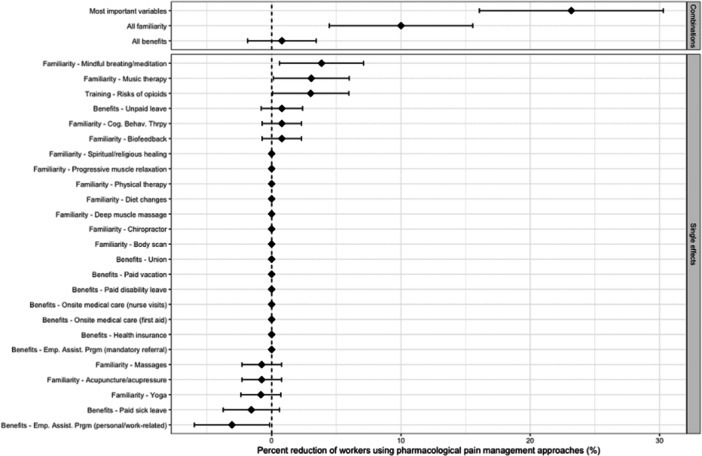
Estimated percent reduction (95% CI) of workers using pharmacological pain management approaches from various counterfactual scenarios. The top three factors indicate simultaneous, multiple counterfactual scenarios, while remaining factors indicate single counterfactual scenarios. An example interpretation is that an estimated 3.9% (95% CI: 0.6%–7.1%) of workers would not use pharmacological pain management approaches if all workers were familiar with mindful breathing/meditation. Note: “most important variables” refers to six variables identified in the Boosted Regression Tree (BRT) model: training on risks of opioids; job benefits for unpaid leave and paid disability; and familiarity with mindful breathing/meditation, with music therapy, and with body scans. “All familiarity” includes all variables which begin with “Familiarity” in the figure and represent familiarity with all nonpharmacological pain management approaches measured in this study. “All benefits” includes all variables which begin with “Benefits” in the figure.

Counterfactually making all workers familiar with mindful breathing/meditation was estimated to produce a 3.9% (95% CI: 0.6%–7.1%) reduction in workers using pharmacological pain management approaches (Figure 2). Familiarity with music therapy or with training on the risks of opioids were also calculated to result in a 3.1% (0.1%–6.0%) and 3.0% (0.1%–6.0%) reduction in workers using pharmacological pain management approaches. No other factors were associated with a statistically significant reduction when their single effect was considered. However, having access to an employee assistance program (EAP) produced a 3.1% (−6.0% to −0.2%) increase in workers using pharmacological pain management approaches. When considering the combination of multiple counterfactual scenarios, the six most important, modifiable variables identified in the BRT model (training on risks of opioids; job benefits for unpaid leave and paid disability; and familiarity with mindful breathing/meditation, with music therapy, and with body scans) were associated with a 23.2% (95% CI: 16.1%–30.3%) reduction estimate. When considering the combination of all familiarity variables, counterfactually making all workers familiar with all nonpharmacological pain management approaches was estimated to reduce pharmacological pain management approaches by 10.0% (4.5%–15.6%). Lastly, when considering the combination of all job benefits, counterfactually providing all workers with the job benefits measured in this study was not significantly associated with a reduction in pharmacological pain management.

**Figure 3:**
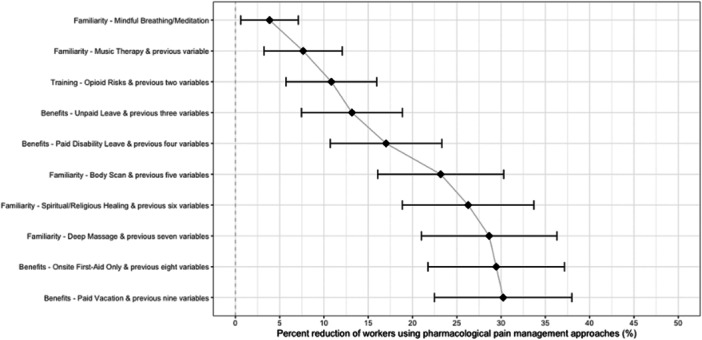
Estimated percent reduction (95% CI) of workers using pharmacological pain management approaches from iterative counterfactual scenarios (from top to bottom). For example, the first counterfactual scenario represents the hypothesized effect of all workers being familiar with mindful breathing/meditation. The second counterfactual scenario represents the hypothesized effect of all workers being familiar with music therapy and the variable above (i.e., familiar with mindful breathing/meditation), and so on. An example interpretation of the third, iterative counterfactual scenario represents the three variables that are estimated to reduce the highest percentage of pharmacological approaches for any combination of three variables. These three variables are familiarity with mindful breathing/meditation, familiarity with music therapy, and training on the risks of opioids, which when combined were associated with a 10.8% (5.7%–16.0%) reduction in pharmacological approaches.

We considered which set of factors may counterfactually reduce the largest percentage of workers using pharmacological pain management approaches (Figure 3). The three strongest factors are familiarity with mindful breathing/meditation, familiarity with music therapy, and training on the risks of opioids, which when combined were associated with a 10.8% (5.7%–16.0%) reduction. Inclusion of five factors (familiarity with mindful breathing/meditation and music therapy, providing unpaid leave and paid disability leave), and, interestingly, training on risks of opioids (which was also a predictor of ever using nonpharmacological pain approaches) was associated with a 17.0% (10.7%–23.3%) reduction and was significantly larger than inclusion of the first factor (familiarity with mindful breathing/meditation; 3.9%, 95% CI: 0.6%–7.1%). Iteratively including each factor shown in Figure 3 was associated with around a 3.5% reduction until the inclusion of the 8th factor (familiarity with deep muscle massages) (total effect: 28.6%, 95% CI: 21.0%–36.3%); no substantial changes were observed past the inclusion of the 8th factor.

## 4 DISCUSSION

Given the high risk of physical injury in construction, many construction workers suffer from MSDs and chronic pain that diminish their well‐being in and out of the workplace. As a result, construction workers have had greater rates of opioid prescription and overdose compared to workers in other industries.^8^, ^25^, ^26^ With the ongoing public health crisis of the U.S. opioid epidemic, largely fueled by the overprescription of opioids to treat pain,^4^, ^27^, ^28^ we sought to explore alternatives via nonpharmacological pain management approaches in the construction industry. The findings from this study may inform evidence‐based training and interventions on chronic pain reduction and pain management among construction workers.

Pharmacological pain management approaches were used slightly more frequently than nonpharmacological pain management approaches among this sample. Only 2% of participants reported using illicit opioids, which may occur after the prescription ended. This is contrast to national data, such as those analyzed from 2007 to 2012, determining that unintentional and undetermined drug‐involved and opioid‐involved overdose deaths attributed to heroin or methadone were highest among construction workers.^29^ Furthermore, most participants reported their musculoskeletal pain made day‐to‐day functioning challenging; nearly three‐quarters attributed the pain to work‐related activities. Guidance from the American Medical Association^30^ and research from pharmacists and healthcare providers^7^, ^27^, ^31^, ^32^ has underscored the negative effects of opioids being overprescribed nationally, both historically and currently, and encouraged using alternatives. While OTCs were the most common pain management strategy reported, of the workers who saw a physician for pain, nearly 1 in 3 (31%) self‐reported that they were still prescribed an opioid by that physician. Other common nonpharmacological approaches reported were use of other substances with the potential for misuse such as alcohol or marijuana, and more widely known approaches, such as ice and heat therapy. This indicates that participants preferred quicker, potentially more easily accessible “numbing” alternatives—especially since many reported lacking job benefits including paid time‐off and coverage for extensive treatment, or were unaware of alternative nonpharmacological pain management strategies.

Of note, female participants had much higher odds of using nonpharmacological approaches within the domains of psychological interventions (CBT), lifestyle changes (changes to diet) and physical relaxation techniques (progressive muscle relaxation, yoga, acupuncture). This is concordant with the literature that indicates women are not only more likely to engage in health seeking behaviors,^33^, ^34^, ^35^ but also be more open to talk therapy^36^, ^37^ and complementary and alternative medicinal (CAM) approaches.^38^, ^39^ Morley and colleagues following over 1000 patients over a 10‐year period to determine how effective CBT, a form of talk therapy, could be for pain management, found that between 14% and 33% of the participants, depending on the outcome measures, achieved clinically significant gains in pain reduction.^40^ Additionally and notably, workers with less experience—who tend to be younger—had higher odds of using CBT, mindful breathing and meditation to address pain, which may be attributed to documented generational differences in acceptance of approaches attitudes toward CAM.^41^, ^42^ Healthcare workers–another worker population that encounters physical and mental exertion albeit at differing levels than construction workers–have self‐reported use of CAM for effective pain management.^43^, ^44^, ^45^ Lastly, we found here that NH Black workers and workers of other ethnicities and races more frequently reported using spiritual/religious healing approaches to manage their pain than did their NH White counterparts. These findings are also supported by literature that indicates the role of spirituality, spiritual beliefs and practices, and spiritual healing are more common among Black, Indigenous and people of color (BIPOC) populations.^46^, ^47^, ^48^, ^49^

From the BRT models, of note, work benefits that allowed for taking unpaid leaves of absence and paid disability were predictors of reduced use of pharmacological approaches. Interestingly, training on the risks of opioids was an important predictor of using pharmacological and nonpharmacological pain management approaches. This dichotomy may be attributed to the fact that we did not inquire about goals of the training, specific training content, training frequency, training modality, or perceived takeaway messages from trainees. Greater worker training around opioid use and opioid use disorder (OUD) prevention (such as asking physicians how to access non‐opioid pain treatment, how to use naloxone, recovery‐friendly workplaces and policies on resources), and ability to take paid leave to allow the body to recovery without fear of job security may decrease potential opioid misuse.^50^, ^51^, ^52^ Our findings of the most important factors that may potentially reduce the use of pharmacological pain management approaches by around 20% among participants was a multi‐faceted approach of mindful breathing/meditation, music therapy, training on the risks of opioids, providing unpaid leave, and providing paid disability leave. Familiarity and use of nonpharmacological pain management approaches by workers included in this study indicates the potential for greater uptake of nonpharmacological pain management in the construction industry. Providing construction workers education on nonpharmacological pain management approaches regardless if they are presently experiencing pain or not, could be part of a long‐term, multifaceted intervention to decrease the burden of pain. The duty of preventing injuries that lead to pain (i.e., primary prevention of MSDs, strains, sprains)^53^ or providing education and training on appropriate pain management approaches and resources should be on employers and the workplace.^7^, ^53^

### 4.1 Practical implications and suggestions

Based on the results of this study, there are a number of implications and suggestions that may be considered by construction industry employers, contractors, and labor unions.

#### 4.1.1 Construction employers

- The utility of occupational safety and health (OSH) efforts, and specifically ergonomic efforts, to prevent the injury‐to‐pain pathway is key. While there is considerable evidence‐based information on how to improve safety and health in construction settings, ultimately the decisions about approaches to improve day‐to‐day OSH conditions onsite are up to the employer/manager.
- It is important that construction employers provide training on pain management, the harms of opioids, and alternatives to pharmacological pain management, as well as reducing the physical work demands on construction workers. Specifically, training content may include topics such as the difference between licit and illicit opioids. the risks of opioid use, how opioid use can become misuse, job benefits for paid and unpaid leave and paid disability for injury and pain, and introducing proven nonpharmacological pain management approaches ^54^, ^55^, ^56^ to their workers.
- Employers should consider changes to workplace policies and coverage. These include but are not limited to: modifications to workers' existing coverage on medical and prescription benefit plans to include nonpharmacological pain management approaches that would otherwise be costly; expansion of coverage, resources, and sessions under EAPs; management support of work‐rest balance, and implementing return‐to‐work programs post‐injury, as light duty can be difficult to accommodate in construction settings.^7^, ^53^, ^57^
- Employers should provide support and educational materials on injury prevention and pain management to workers in a language they will understand and that is culturally appropriate.

#### 4.1.2 Labor unions

- Labor unions already prioritize open, frequent, and transparent communication with their members on a number of work‐related issues. Moreover they serve as champions for the workplace safety, health, and well‐being of their members.^58^, ^59^, ^60^ North America's Building Trades Unions' (NABTU) Resolution No. 4 focuses on “Support Efforts to Reduce Pain, Opioid Use, Opioid Overdose and the Number of Deaths by Suicide in the Construction Agency.”^61^ NABTU and all Building Trades Councils fully endorse interventions such as working to destigmatize substance use and mental health disorders through culturally and linguistically appropriate services, education, and awareness with members, leadership, and owners; mandating all apprentices and trainees to complete a training program to increase awareness of work‐related injuries associated with opioid use; supporting Naloxone training for members; and encouraging organizations to develop and support workplace policies and programs that promote rehabilitation and return‐to‐work and recovery‐friendly workplaces.^61^
- Labor unions could also provide training on how to prevent MSDs, managing chronic pain (including nonpharmacological pain management approaches), the risks of opioids, and work benefits that allowed for paid and unpaid leave of absences. Labor unions can also have a critical role in supporting their members through the process of requesting paid disability and workers' compensation, if required.^61^, ^62^, ^63^
- Labor unions should consider using peer advocacy programs that use trained members or peers to assist others in accessing services for substance use and mental health disorders and to support members after they have returned to work following treatment. For example, CPWR has a resource titled, “Peer Advocacy for Construction Workers Struggling with Substance Use and Mental Health” that discusses peer advocacy in great detail and provides an interview instrument to better understand the union's reactions toward peer advocacy around the opioid epidemic, and specific interventions.^64^
- Sharing information through labor union channels about the scope of the problem of opioids in the workplace and recovery‐friendly workplaces with rank‐and‐file trades so they can better understand and support fellow union members and colleagues experiencing OUD.^65^, ^66^, ^67^, ^68^

#### 4.1.3 Occupational and environmental health and safety (OEHS) professionals

- OEHS professionals embedded in workplaces or contracting for employers should mitigate or eliminate work‐site hazards to alleviate MSDs and other workplace injuries that may lead to strain and pain. From simple measures such as conducting job hazard analysis, ensuring a proper level of staffing, and using prevention through design techniques to mitigate or eliminate hazards, OEHS professionals can take steps to reduce workplace injuries and subsequent opioid use by workers.
- OEHS professionals should facilitate workplace education and training on managing pain and uptake of targeted interventions, in conjunction with engineering and administrative controls to reduce workplace MSDs and strains that cause pain.

### 4.2 Limitations

There are several limitations to consider when interpreting the findings of this study. First, the data from this pilot study were cross‐sectional; therefore, causality cannot be inferred – in other words, we are not saying that nonpharmacological approaches prevent opioid overdose or addiction but that associations were demonstrated between nonpharmacological approaches and our examined outcomes. Second, while reCAPTCHA was used to ensure no bots or AI programs participated in the survey, we did not follow‐up with participants to ensure that they were indeed construction workers, since survey participation was anonymous and did not collect any personal identifiers. Third, as this was a voluntary, self‐reported survey, bias may have been introduced—especially self‐selection and social desirability bias. Fourth, self‐reported treatment for pain was not cross‐validated with medical records. Fifth, the survey was only conducted in English so those with limited English comprehension who use nonpharmacological pain management approaches were not represented in the sample. Sixth, as mentioned in the Methods section, this is a small convenience sample given the lack of study funding to incentivize participation. Lastly, we did not ask the participants for their geographic location so the impact on access to nonpharmacological pain management approaches is unknown and therefore may not be generalizable to the entire U.S. construction workforce.

## 5 CONCLUSIONS

To our knowledge, this is the first study examining the familiarity and utilization of nonpharmacological pain management among U.S. construction workers. Chronic pain is challenging and can lead to other adverse health conditions including opioid misuse and overdoses. Unfortunately, workers in the construction industry have been disproportionately affected by the ongoing public health crisis of the opioid epidemic. The study findings show that construction workers were more familiar with certain nonpharmacological approaches to pain management (OTCs, physical therapy) than others such as biofeedback, progressive muscle relaxation, and body scans. Important factors to potentially reduce reliance on prescription opioids for pain management involve a multi‐faceted intervention of engineering controls, administrative controls, and integrating nonpharmacological pain management approaches. All in all, the findings from this study can help to better inform workplaces of methods to reduce pain among construction workers without the use of prescription opioids.

## AUTHOR CONTRIBUTIONS

ABL: Conceptualization, methodology, investigation, writing—original draft, writing—review & editing; AS: Formal analysis, data curation, visualization, writing—original draft, writing—review & editing; SE: Conceptualization, supervision, project administration, funding acquisition, writing—review & editing; EG: Conceptualization, resources, writing—review & editing; DT: Conceptualization, resources, writing—review & editing; SDC: Conceptualization, methodology, investigation, writing—review & editing. All authors have given final approval of this version to be published and agree to be accountable for all aspects of the work ensuring that questions related to the accuracy or integrity of any part of the work are appropriately investigated and resolved.

## CONFLICTS OF INTEREST STATEMENT

The authors declare that there are no conflicts of interest.

## DISCLOSURE BY AJIM EDITOR OF RECORD

John Meyer declares that he has no conflict of interest in the review and publication decision regarding this article.

## ETHICS APPROVAL AND INFORMED CONSENT

This study was approved and deemed exempt by the University of Michigan Institutional Review Board on August 17, 2022, Protocol # HUM00222058.

## DISCLAIMER

This study represents the work and view solely of the authors and does not necessarily represent the views or endorsement of the Centers for Disease Control and Prevention (CDC)/National Institute for Occupational Safety and Health (NIOSH).

## Supporting information

Supporting information.

## Data Availability

The data that support the findings of this study are available from the corresponding author upon reasonable request.
