# Supplementary material for: Nonpharmacological pain management approaches among U.S. construction workers: A cross‐sectional pilot study
Source: Am J Ind Med. 2024 Jun 20;68(Suppl 1):S158–70. doi: 10.1002/ajim.23630 (PMC11869802; doi:10.1002/ajim.23630)
Supplement: Supplementary file 1 — Supporting information. [file AJIM-68-S158-s001.docx]

**APPENDICES**


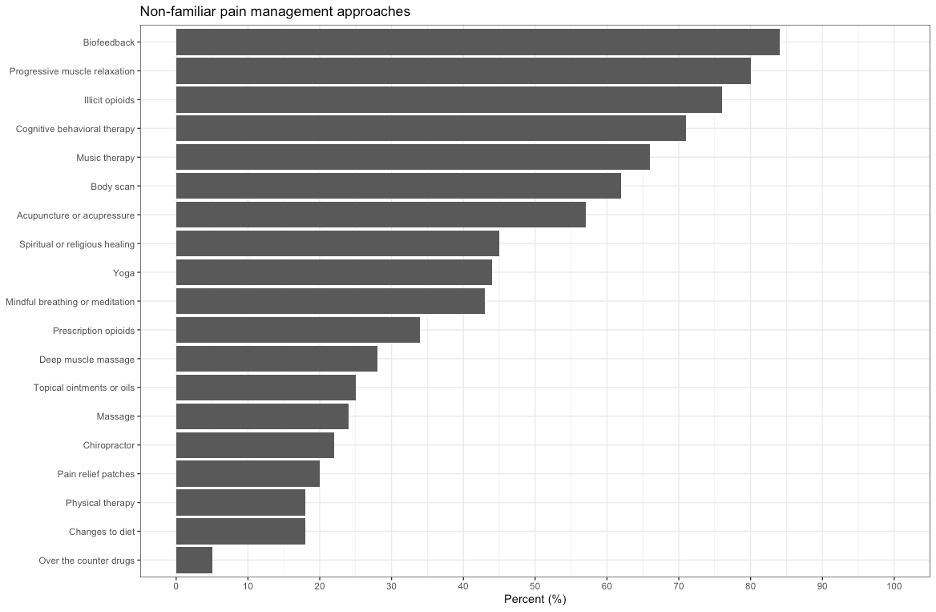


**FIGURE A1** Percentage of respondents who self-reported they were not familiar with a pharmacological or non-pharmacological pain management approach. Note that this is the percentage of participants who said that they were “Not at all familiar” with the given pain management approach.

| **TABLE A1**: Relative importance of 37 variables included boosted regression model of ever using pharmacological pain management approaches (n = 129) | |
| --- | --- |
| **Variable** | **Relative Importance** |
| Pain made work/home activities difficult | 19.6% |
| Education level | 9.1% |
| Familiarity with prescription opioids | 6.8% |
| Years work experience | 6.2% |
| Training on risks of opioids | 5.8% |
| Seen physician for pain | 5.3% |
| Familiarity with mindful breathing/meditation | 4.3% |
| Benefits – unpaid leave | 3.9% |
| Familiarity with music therapy | 3.9% |
| Familiarity with topical ointments/oils | 3.6% |
| Familiarity with body scans | 3.5% |
| Benefits – paid disability leave | 3.2% |
| Benefits – employee assistance program (personal) | 2.7% |
| Familiarity with acupressure/acupuncture | 2.6% |
| Familiarity with spiritual/religious healing | 2.2% |
| Familiarity with yoga | 2.1% |
| Benefits – onsite medical care (first aid only) | 2.1% |
| Benefits – paid vacation | 2.1% |
| Familiarity with pain relief patches | 2.0% |
| Familiarity with cognitive behavioral therapy | 1.7% |
| Familiarity with illicit opioids | 1.4% |
| Familiarity with deep muscle massages | 1.2% |
| Benefits – paid sick leave | 1.0% |
| Familiarity with massages | 0.9% |
| Ever used non-pharmacological pain management approaches | 0.9% |
| Benefits – employee assistance program (mandatory referral only) | 0.7% |
| Familiarity with progressive muscle relaxation | 0.7% |
| Familiarity with biofeedback | 0.2% |
| Gender | 0.2% |
| Benefits – union | 0.1% |
| *Note: Seven variables had a relative importance of 0%: (1) Benefits – health insurance; (2) Benefits – nurse visit; (3) Used over-the-counter drugs; (4) Familiarity with chiropractors; (5) Familiarity with changes to diet; (6) Familiarity with physical therapy; and (7) ever experienced pain/discomfort in body in the last year.* | |
